# Factors associated with dengue prevention behaviour in riverbank area: A cross-sectional study

**DOI:** 10.1016/j.amsu.2021.102450

**Published:** 2021-06-02

**Authors:** Firdian Makrufardi, Paulin Surya Phillabertha, Erri Larene Safika, Sungkono

**Affiliations:** a Faculty of Medicine, Public Health and Nursing, Universitas Gadjah Mada/Dr. Sardjito Hospital, Yogyakarta, Indonesia; b Department of Education Technology, Faculty of Education Science, Universitas Negeri Yogyakarta, Yogyakarta, Indonesia

**Keywords:** 3M practices, Dengue prevention, Socio-demographic, Riverbank

## Abstract

**Introduction:**

Dengue has been a burden, especially in tropical country. Indonesian Ministry of Health promote dengue prevention through environmental control with 3 M (covering water storage, cleaning water storage, and recycling unused items) practices. Here we analyzed factors associated with dengue prevention behavior in riverbank area of Yogyakarta Province.

**Methods:**

The study employed cross-sectional survey covering riverbank area of Sendowo, Yogyakarta Province. This study systematically sampled 1 representative from every household to describe the condition of 1 house. We analyzed the associations between subjects' characteristics and dependent variables using Pearson's chi-square test.

**Results:**

Overall, 89 subjects were enrolled in this study, of whom 11.2% males and 88.8% females. Age and occupation were associated with covering water storage variable with p-value of <0.001 and 0.007, respectively. Recycling unused items variable was associated with monthly income with p-values of 0.045. Furthermore, there were no significant associations between cleaning water storage variable with sex, age, marital status, education level, monthly income, and occupation.

**Conclusion:**

Age and occupations were associated with covering water storage variable. Recycling unused items variable shows significant association with income. Further multiarea study is necessary to compare our findings with other areas.

## Highlights

• Dengue has been a burden in tropical country
• Several factors associated with dengue prevention behavior in riverbank area
• It is essential to promote dengue prevention through environmental control

## 1 Introduction

According to World Health Organization, Dengue is a virus that transmitted by female mosquito *Aedes aegypti.* Its bites cause infection in human and manifest from Dengue Fever (DF), Dengue Haemorrhagic Fever (DHF), and Dengue Shock Syndrome (DSS). The Dengue Virus (DENV) belong to genus *Flavivirus* and family *Flaviviridae*. It consists of 4 serotype virus as DENV-1, DENV-2, DENV-3, and DENV-4 [1,2].

Indonesia Ministry of Health reported that Indonesia had 68,407 dengue cases in 2017 with Province of West Java, East Java, and Central of Java as the 3 provinces with highest cases. The Incidence Rate (IR) of dengue cases decreased dramatically from 78.85 per 100,000 populations into 26.12 per 100,000 populations in 2017. The IR of dengue cases in Yogyakarta Province was 43.65 per 100,000 populations and the case fatality rate (CFR) was 0,43 [3,4]. Sleman district contributed 603 new cases with 2 of them being the death cases in June 2020. Sub-districts in Sleman that contribute to the highest cases are Mlati, Godean, Gamping, Ngaglik, Depok, and Sleman. Sendowo is a riverbank area in Mlati sub-district, Sleman [[7], [9]]. It is known that the location of residence around the river with a buffer of 0–100 m and 100–1000 m have an effect on the incidence of dengue infection. The results of spatial analysis conducted in Bantul district showed that the houses where residents live at this distance have a higher incidence of dengue infection [8].

To prevent the transmission of dengue virus, there are 2 basic ways namely eradicating mosquito nests and eradicating the adult mosquito. There are 3 types of basic control to prevent and control to eradicate mosquito nests, such us environmental control, biology control, and chemical control. Indonesian Ministry of Health promote dengue prevention through environmental control with 3 M program. The 3 M program is carried out by draining places that used as water reservoirs minimum once a week (CL), closing tightly the water reservoirs (CO), and recycling unused items that have the potential to become water reservoir (RI). The purpose of this program prevents the breeding of mosquitos and laying eggs [3,5].

There are several factors that affect the success of 3 M Program. Previous research that conducted in Banjarbaru City revealed that knowledge have relationship with community action to eradicate mosquito nest in endemic area and community education have significant relationship with the presence of larvae *Ae. aegypti*. Research that conducted in Blora district revealed that several factors like age, gender, education background, and money income didn't have relationship with the success of 3 M Program, but it may be varied in other region in Indonesia [5,6].

This study was conducted to identify socio-demographic factors that associate with dengue prevention practices in riverbank area of Yogyakarta Province.

## 2 Methods

### 2.1 Participant and data collection

Sendowo sub-district is a densely populated region with diverse demographic and socioeconomic indicators in Yogyakarta province, Indonesia that is suitable for the study criteria. The location of the Sendowo sub-district which is on the eastern part of the Code river provides an overview of the conditions of housing and residents who live in the riverbank of Yogyakarta province. This study systematically sampled 1 representative from every household to describe the condition of 1 house. This was done to avoid concentration of respondents in few houses and to ensure fair distribution of sample across districts.

The study employed cross-sectional survey. The participants were recruited during a community service program. The potential participants were 89 and recruited by posting an announcement on the bulletin board, and inviting participants through local leader. This free consultation is a collaboration between the Directorate of Community Service Universitas Gadjah Mada and Faculty of Medicine, Public Health and Nursing Universitas Gadjah Mada during student community service program (Inspired Bulaksumur Urban Community) in April 2019. This study consecutively took all subjects during community service programs held in Sendowo district. The pediatric population (0 to <18 years) was withdrawn from this study. Upon informed and voluntary consent, the subjects answered the questionnaire package. A structured questionnaire were cross-sectionally used to collect field data.

We identified the subjects' age based on birth date in their ID card. Sex is classified based on males and females. The level of education was defined as the completed grade of schooling. The income of household per month, consisting of both cash and kind from all sources within the month was used to defined income level. We defined marital status as being single or married. The subject who experiences divorce was deemed as single. The operational definition for the dependent variable was based on the Indonesian Ministry of Health dengue prevention practices (Table 2). The dependent variable “3 M program” including covering water storage (CO), cleaning water storage (CL), and recycling unused items (RI).^3,5^ This study has been reported in line with the STROCSS criteria [28].

### 2.2 Data analysis

Descriptive statistics were carried out to describe the socio-demographic characteristics of the study sample. Data were analyzed using IBM SPSS Statistics 23rd version (IBM Corp., Chicago). The associations between subjects' characteristics and dependent variables were determined using Pearson's chi-square test, with p-value of <0.05 considered as significant.

### Ethics approval

This study was approved by the Ethics Committee of the Faculty of Medicine, Public Health, and Nursing, Universitas Gadjah Mada, Yogyakarta, Indonesia.

## 3 Results

### 3.1 Baseline characteristics

Overall, there were 11.2% of males and 88.8% of females. Subjects were dominated by adults age between 35 and 60 years (77.5%). Of 89 subjects, 74 subjects have the status of being married. Almost half of the subjects have education up to senior high school (43.8%), and about 19.1% was followed by junior high school. A total of 45 subjects earned between 2.5 million rupiahs to 5 million rupiahs. The most common occupation was housewife. Other reported occupations were entrepreneur/trader (13.5%), private employees (7.9%), fisherman (5.6%), laborer (4.5%), does not work (3.4%), student (2.2%), service provider (2.2%), government employees (2.2%), and others (1.1%).

Of 89 subjects, about two-third of subjects were always covering water storage (67.4%). Almost half of the subjects (44.9%) once a week cleaning water storage. About 66.3% of subjects recycled used goods at least once a week, including 16.9% once a week, 29.2% twice a week, and 20.2% three or more times a week (Table 1).

**Table 1:** Operationalization and coding of dengue prevention variables.

Variables	Operational definition	Category	Code	Freq.	%
Covering water storage	Close tightly water reservoirs such as bathtubs and drums. Closing can also be interpreted as the activity of burying used goods in the ground	Do not know	1	1	1.1
		Never	2	19	21.3
		Usually (1–6 times a week)	3	9	10.1
		Always (7 times a week)	4	60	67.4
Cleaning water storage	Clean/drain places that are often used as water reservoirs such as bathtubs, jugs, water toren, drums and other water reservoirs.	Do not know	1	2	2.2
		Never	2	27	30.3
		Once a week	3	40	44.9
		Twice a week	4	18	20.2
		Three or more times a week	5	2	2.2
Recycling items	Recycling used goods that are economically valuable (recycled)	Do not know	1	18	20.2
		Never	2	12	13.5
		Once a week	3	15	16.9
		Twice a week	4	26	29.2
		Three or more times a week	5	18	20.2

Source: authors' own construct, 2019.

**Table 2:** Association between characteristics and covering water storage variable.

Characteristics	Freq.	%	Code of covering water storage variable, N				p
			1	2	3	4	
Sex							
Male	10	11.2	0	1	1	8	0.784
Female	79	88.8	1	18	8	52	
Age							
Young (<35 years old)	15	16.9	0	3	4	8	<0.001
Middle (35–60 years old)	69	77.5	0	16	4	49	
Elderly (>60 years)	5	5.6	1	0	1	3	
Marital status							
Single	15	16.9	1	4	0	10	0.072
Married	74	83.1	0	15	9	50	
Education level							
Not attending school	11	12.4	1	4	1	5	0.261
Elementary school	15	16.9	0	3	0	12	
Junior high school	17	19.1	0	4	0	13	
Senior high school	39	43.8	0	7	7	25	
Bachelor degree	3	3.4	0	0	0	3	
Master/Phd	4	4.5	0	1	1	2	
Monthly income							
≤1 million rupiahs	1	1.1	0	0	0	1	0.764
1–2.5 million rupiahs	36	40.4	1	8	6	21	
2.5–5 million rupiahs	45	50.6	0	11	2	32	
5–7.5 million rupiahs	5	5.6	0	0	1	4	
>7.5 million rupiahs	2	2.2	0	0	0	2	
Occupation							
Does not work	3	3.4	0	1	0	2	0.007
Student	2	2.2	0	0	0	2	
Housewife	51	57.3	1	11	4	35	
Government employees	2	2.2	0	1	0	1	
Private employees	7	7.9	0	2	5	0	
Entrepeneur/trader	12	13.5	0	1	0	11	
Service provider	2	2.2	0	0	0	2	
Fisherman	5	5.6	0	0	0	5	
Laborer	4	4.5	0	3	0	1	
Others	1	1.1	0	0	0	1	

Source: authors' survey, 2019.

### 3.2 Association between socio-demographic characteristics and variables

We found that age and occupation were associated with covering water storage variable with p-value of <0.001 and 0.007, respectively. Sex, marital status, education level, and monthly income were not associated with covering water storage variables (Table 2). Recycling unused items variable was associated with monthly income with p-values of 0.045. No association was found between recycling unused items variable and other characteristics (Table 3). Furthermore, there were no associations between cleaning water storage variable with sex, age, marital status, education level, monthly income, and occupation with p-values >0.05 (Table 4).

**Table 3:** Association between characteristics and cleaning water storage variable.

Characteristics	Freq.	%	Code of cleaning water storage variable, N					p
			1	2	3	4	5	
Sex								
Male	10	11.2	0	3	5	2	0	0.784
Female	79	88.8	2	24	35	16	2	
Age								
Young (<35 years old)	15	16.9	0	5	5	5	0	0.886
Middle (35–60 years old)	69	77.5	2	21	32	12	2	
Elderly (>60 years)	5	5.6	0	1	3	1	0	
Marital status								
Single	15	16.9	0	7	5	3	0	0.966
Married	74	83.1	2	20	35	15	2	
Education level								
Not attending school	11	12.4	0	4	6	1	0	0.929
Elementary school	15	16.9	0	3	8	4	0	
Junior high school	17	19.1	1	4	8	3	1	
Senior high school	39	43.8	1	12	15	10	1	
Bachelor degree	3	3.4	0	1	2	0	0	
Master/Phd	4	4.5	0	3	1	0	0	
Monthly income								
≤1 million rupiahs	1	1.1	0	0	1	0	0	0.965
1–2.5 million rupiahs	36	40.4	0	11	16	8	1	
2.5–5 million rupiahs	45	50.6	2	15	18	9	1	
5–7.5 million rupiahs	5	5.6	0	0	4	1	0	
>7.5 million rupiahs	2	2.2	0	1	1	0	0	
Occupation								
Does not work	3	3.4	0	1	2	0	0	0.966
Student	2	2.2	0	0	2	0	0	
Housewife	51	57.3	1	12	25	11	2	
Government employees	2	2.2	0	2	0	0	0	
Private employees	7	7.9	0	2	3	2	0	
Entrepeneur/trader	12	13.5	1	3	5	3	0	
Service provider	2	2.2	0	2	0	0	0	
Fisherman	5	5.6	0	3	1	1	0	
Laborer	4	4.5	0	2	1	1	0	
Others	1	1.1	0	0	1	0	0	

Source: authors' survey, 2019.

**Table 4:** Association between characteristics and recycling unused items variable.

Characteristics	Freq.	%	Code of recycling unused items variable, N				p
			1	2	3	4	
Sex							
Male	10	11.2	2	8	0	0	0.489
Female	79	88.8	10	54	1	14	
Age							
Young (<35 years old)	15	16.9	1	9	0	4	0.611
Middle (35–60 years old)	69	77.5	11	47	1	10	
Elderly (>60 years)	5	5.6	0	5	0	0	
Marital status							
Single	15	16.9	1	12	0	2	0.761
Married	74	83.1	11	50	1	12	
Education level							
Not attending school	11	12.4	1	9	0	1	0.630
Elementary school	15	16.9	0	14	0	1	
Junior high school	17	19.1	1	12	0	4	
Senior high school	39	43.8	8	23	1	7	
Bachelor degree	3	3.4	1	1	0	1	
Master/Phd	4	4.5	1	3	0	0	
Monthly income							
≤1 million rupiahs	1	1.1	0	1	0	0	0.045
1–2.5 million rupiahs	36	40.4	7	24	0	5	
2.5–5 million rupiahs	45	50.6	4	32	0	9	
5–7.5 million rupiahs	5	5.6	1	3	1	0	
>7.5 million rupiahs	2	2.2	0	2	0	0	
Occupation							
Does not work	3	3.4	0	3	0	0	0.988
Student	2	2.2	0	2	0	0	
Housewife	51	57.3	8	31	1	11	
Government employees	2	2.2	0	2	0	0	
Private employees	7	7.9	2	3	0	2	
Entrepeneur/trader	12	13.5	1	10	0	1	
Service provider	2	2.2	0	2	0	0	
Fisherman	5	5.6	1	4	0	0	
Laborer	4	4.5	0	4	0	0	
Others	1	1.1	0	1	1	0	

Source: authors' survey, 2019.

## 4 Discussion

This study reveals that age and occupations were associated with CO variable. RI variable shows significant association with income. We found no associations between socio-demographic characteristics and CL variable.

This is a novel community-based study that was conducted to describe the association between socio-demographic characteristics and dengue prevention practices in a riverbank area in Indonesia. These results are discussed bearing in mind that this study has certain limitations. Firstly, this is a cross-sectional study, so we couldn't describe the process or effects one variable have on the others. Second, this study is conducted in one riverbank area in Yogyakarta, so it can't be generalized to other riverbank areas in Indonesia.

In present study, age was associated with CO practice. The middle age group have relatively higher frequency of the practice than other age group. This is consistent with earlier studies in Malang, Indonesia [21] and Malaysia [10]. It is possible because older people had more experience with dengue and got more information about preventive practice from health workers, cadres, or posyandu (integrated healthcare) [21].

We found association between occupation and CO practice. Housewife was found to do CO more often. This could happen because women, mostly housewives in Indonesia have more social role of caring the household and community [20]. The responsibility of the health of the entire family rely on women. Moreover, members of PKK (Women Empowerment Welfare Group), mostly housewifes, engages in a lot of social community activites, including delivering dengue prevention information [21,24]. Interestingly, we found no association between sex and CO.

Current study also found association between RI practice and monthly income, but no association with other socio-demographic characteristics. Another study found significant relationship between family income and dengue prevention practices, but didn't specifically mention the RI practice [14,22].

It is possible that recycling unused items provide positive impact on family income in current setting. Most of the respondents’ monthly income are around Sleman minimum wage at 1.8 million rupiah (131 USD), and recycling could be an alternative to minimize family expenditure or increase it by selling the unused goods. In low-to-middle-income countries, recycling is seen as a private economic activity based on valorisation and trading. Unlike in high-income counterparts, recycling aims to extract any remaining economic values of unused items, to prevent removal, and commercialise them through accumulating, eliminating contamination, sorting, storage, and marketing [23].

Household waste is associated with *Aedes* sp breeding that can increase the risk of dengue infection. Porcelaine and plastic wastes are found more in urban areas [13]. Indonesian Ministry of Health's recommendation is to recycle any unused items that still have economic value or have potential as a breeding sites for mosquitos. This way could limit the addition of waste to the environment and reduce the proliferation of dengue vector [11]. A study in Banjarbaru, South Kalimantan found that recycling used goods was associated with the incidence with dengue [18].

We found no association between CO practice and education level, marital status, and monthly income. Also, we didn't find any association between CL practice and socio-demographic characteristics. In general, most participants from all different educational background have good CO and CL practice (always or usually close and clean water storage). Formal education had roles in introducing dengue prevention to students [15]. Knowledge on dengue prevention is associated with water container protection practices [[16], [26]].

Indonesian Ministry of Health issued a law concerning the National DF/DHF Prevention and Control Programme in 1992, with commitment and participation from Ministry of Home Affairs, Ministry of Education, Women Empowerment Welfare Group (PKK), and cadres. Around the following year, dengue prevention practice was introduced in schools. Since 2000, Indonesian health authorities has been more focused on empowering the community on dengue prevention through Jumantik cadres (larva monitoring workers) and PKK. They act as spearheads of the control of dengue vectors by giving basic health education and door-to-door visits for promoting and implementing dengue prevention such as covering, cleaning, and recycling unused items that have potential as breeding sites (3 M) [17].

It is possible that Jumantik cadres and PKK are another resource of information about dengue prevention practice in this area. Social organization thrive well in densely populated region in Yogyakarta like Sendowo district and could be more effective in spreading information about the importance of healthy living [27].

A case in Bali shows the importance of Jumantik cadres in reducing DF/DHF cases. In 2011, Bali was one with the highest cases of DF/DHF in Indonesia. Jumantik cadres roles was to monitor the community and environment in Denpasar, Bali. Its main focus was densely populated urban areas with high mobility. These caders were paid monthly by the regional government who provided greater funding for the program. After 2 years, the cases drop remarkably [19].

Most participants in this study are women, and the majority are housewives. A study in Kalasan sub-district reported that most Jumantik cadres are women and recruited through PKK. Only a few men participated as Jumantik cadres [25]. It is possible that in current region, the health promoting activities including dengue prevention are mostly handled by women.

Dengue prevention practice should be the responsibility of all genders. A study in six Asian countries shows that the majority of reported dengue incidences involve males [12]. Another study in Malang shows that more male respondents have poor dengue prevention behavior compared with female [21]. Male should be more encouraged to participate in dengue prevention activities so both male and female have roles in protecting their society from dengue.

Findings in this study encourage local governments to support any local communities in dengue prevention programs. Forming a specific group or empowering available community in the area on dengue prevention practices could be beneficial in decreasing dengue cases. Communities should be educated about specific practices to inhibit the reproduction of *Aedes* sp from school-age. Different riverbank areas are likely to show different patterns in dengue prevention practices due to the uniqueness on the environments, communities, customs, regulations, or others. It is important to find out factors associated with dengue prevention practices in different riverbank areas in Indonesia to figure out the different patterns on the practices.

## 5 Conclusion

Age and occupations were associated with covering water storage variable. Recycling unused items variable shows significant association with income. Further multiarea study is necessary to compare our findings with other areas.

## Provenance and peer review

Not commissioned, externally peer-reviewed.

## Consent statement

Written informed consent was obtained from all of the patients for publication of study. A copy of the written consent is available for review by the Editor-in-Chief of this journal on request.

## Ethical approval

This study was approved by the Ethics Committee of the Faculty of Medicine, Public Health, and Nursing, Universitas Gadjah Mada, Yogyakarta, Indonesia.

## Funding sources

The authors declare that this study had no funding source.

## Authors’ contribution

Firdian Makrufardi conceived the study, drafted the manuscript. Paulin Surya Phillabertha and Erri Larene Safika drafted the manuscript, and Sungkono critically revised the manuscript for important intellectual content. All authors facilitated all project-related tasks.

## Registration of research studies

ISRCTN75270383.

## Guarantor

Firdian Makrufardi.

## Declaration of competing interest

The author(s) declared no potential conflicts of interest with respect to the research, authorship, and/or publication of this article.
